# China National Medical Products Administration approval summary: anlotinib for the treatment of advanced non-small cell lung cancer after two lines of chemotherapy

**DOI:** 10.1186/s40880-019-0383-7

**Published:** 2019-06-20

**Authors:** Ming Zhou, Xiaoyuan Chen, Hong Zhang, Lin Xia, Xin Tong, Limin Zou, Ruimin Hao, Jianhong Pan, Xiao Zhao, Dongmei Chen, Yuanyuan Song, Yueli Qi, Ling Tang, Zhifang Liu, Rong Gao, Yuankai Shi, Zhimin Yang

**Affiliations:** 1 Medical Review Department 1, Center for Drug Evaluation, China National Medical Products Administration, No. 128 Jianguo Road, Chaoyang District, Beijing, 100022 P. R. China; 2 Center for Drug Inspection, China National Medical Products Administration, Beijing, 100037 P. R. China; 3 Department of Medical Oncology, Beijing Key Laboratory of Clinical Study on Anticancer Molecular Targeted Drugs, National Cancer Center/National Clinical Research Center for Cancer/Cancer Hospital, Chinese Academy of Medical Sciences & Peking Union Medical College, Beijing, 100021 P. R. China

**Keywords:** Advanced non-small cell lung cancer, Anlotinib, Anti-angiogenesis, Epidermal growth factor receptor, Activating anaplastic lymphoma kinase, Adverse drug reaction, National Medical Products Administration

## Abstract

**Background:**

On May 8, 2018, the China National Medical Products Administration (NMPA) approved anlotinib, an orally administered anti-angiogenesis inhibitor, for the treatment of patients with advanced non-small cell lung cancer (NSCLC) who have progressed after treatment with two or more lines of prior systemic chemotherapy.

**Main body of the abstract:**

China NMPA reviewed and inspected a regional double-blinded, placebo-controlled, Phase III trial comparing the overall survival (OS) of NSCLC patients between the anlotinib and placebo arms. A total of 437 patients were randomized (2:1) to receive either anlotinib (*n* = 294) or placebo (*n* = 143) once daily on a 2-week on and 1-week off schedule. Patients with epidermal growth factor receptor (*EGFR*) or activating anaplastic lymphoma kinase (*ALK*) genomic tumor aberrations should have disease progression on NMPA-approved therapy. Anlotinib is the first NMPA-approved drug for patients with advanced NSCLC who have progressed on at least two lines of prior systemic chemotherapies in China. The approval was based on a statistically and clinically significant improvement in median OS with anlotinib (9.46 months) compared with placebo [6.37 months; hazard ratio (HR]) = 0.70, 95% confidence interval (CI) = 0.55–0.89; two-sided log-rank *P* = 0.002]. The confirmed objective response rate (ORR) was 9.2% in the anlotinib arm and 0.7% in the placebo arm. The median duration of response (DoR) was 4.83 months, with a 95% CI of 3.31–6.97 months. The toxicity profile of anlotinib was consistent with that of known anti-angiogenesis inhibitors. Common adverse drug reactions (ADRs) in anlotinib-treated patients included hypertension (67.4%), hand–foot syndrome (43.9%), hemoptysis (14.0%), thyroid stimulating hormone (TSH) elevation (46.6%), and corrected QT interval (QTc) prolongation (26.2%).

**Short conclusion:**

Anlotinib demonstrated a clinically significant OS prolongation as a novel therapeutic option for advanced or metastatic NSCLC following at least two lines of chemotherapy.

## Background

Lung cancer is the leading cause of death among all malignancies [1], accounting for approximately 6,102,000 deaths annually in China [2]. Most lung cancers are non-small cell lung cancer (NSCLC) and usually diagnosed at an advanced stage with a low survival rate. Over the last decade, overall survival (OS) of patients with advanced NSCLC has been significantly prolonged due to the use of targeted therapies, including tyrosine kinase inhibitors (TKIs) [3–5] and immune checkpoint inhibitors [6–8]. However, patients who progressed after targeted therapy and patients with wild-type driver genes after systemic treatment still have poor prognosis.

At present, there are no standard therapies approved by The China National Medical Products Administration (NMPA, formerly known as China Food and Drug Administration [CFDA]) for patients with advanced NSCLC who have progressed after treatment with approved targeted therapies (e.g., TKIs) or at least two lines of chemotherapy.

In recent decades, anti-angiogenesis therapy has become a crucial strategy for the treatment of solid tumors, such as hepatocellular carcinoma [9], colorectal carcinoma [10], and NSCLC [11]. In 2014, the European Medicines Agency (EMA) approved nintedanib, the first TKI targeting vascular endothelial growth factor receptor (*VEGFR*), platelet-derived growth factor receptor (*PDGFR*), and fibroblast growth factor receptor (*FGFR*), for the treatment of advanced NSCLC in combination with docetaxel. This approval was based on a 2-month OS benefit from the LUME-Lung 1 study [12]. However, nintedanib was not approved by the United States Food and Drug Administration (U.S. FDA). The LUME-Lung 2 study (nintedanib plus pemetrexed vs. placebo plus pemetrexed) failed to demonstrate an OS benefit [13]. Several anti-angiogenesis inhibitors, such as sorafenib [14], sunitinib [15], and vandetanib [16], reached a Phase III confirmatory trial but still failed to win approval, either as monotherapy or in combination with chemotherapy. Since there were no standard treatments for patients with NSCLC who have progressed after at least two systemic therapies in China, there is significant unmet medical need in these cancer patients.

Anlotinib (AL3818), developed by Chia Tai Tianqing Pharmaceutical Group Co., Ltd. (CTTQ; Lianyungang, Jiangsu, China), is an orally administered TKI that targets *VEGFR1*-*3*, *FGFR1*-*4*, *PDGFRαGβ*, rearranged during transfection (*RET*), and stem cell factor receptor (also known as *c*-*Kit*). It showed promising anti-cancer activities both in vitro and in vivo [17]. In March 2011, CTTQ was authorized by CFDA (now NMPA) to conduct a Phase I/II clinical trial for anlotinib, which had shown that anlotinib at 12 mg once daily on a 2-week on and 1-week off schedule displayed manageable toxicity profiles. Dose-limiting toxicities (DLTs) at the dose of 16 mg were Grade 3 hypertension and fatigue [18]. In March 2015, after the Phase II trial (ALTER0203) for advanced NSCLC has been completed, CTTQ initiated a Phase III clinical study (ALTER0303) in China after discussion with the Center for Drug Evaluation (CDE) of NMPA.

### Trial design

**Fig. 1:**
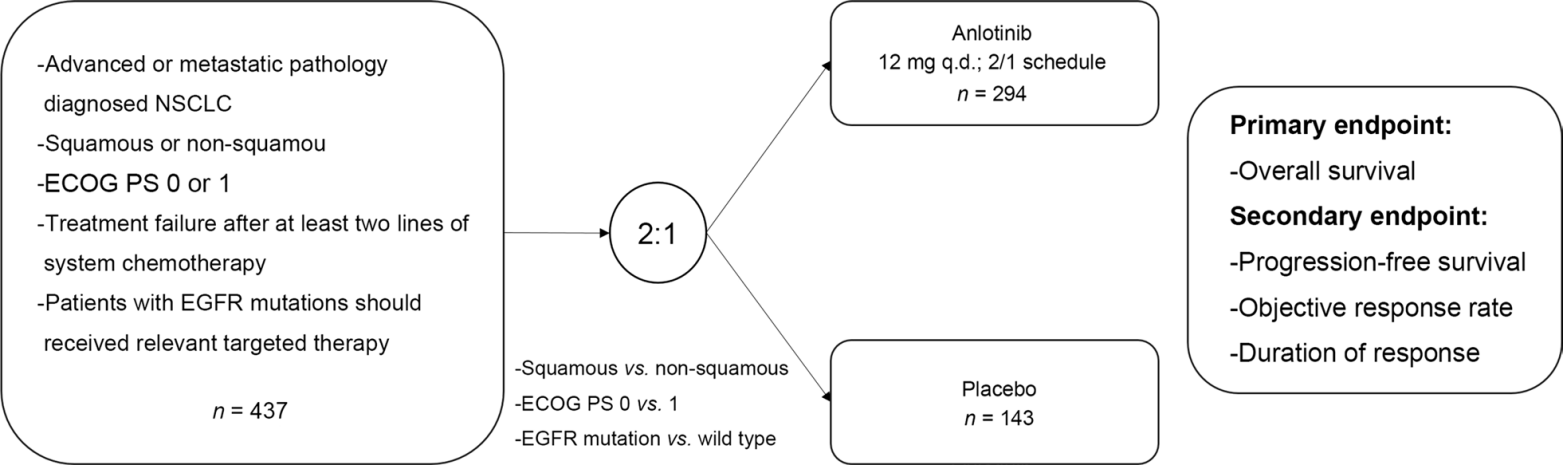
Study design of the ALTER0303 trial. NSCLC, non-small cell lung cancer; ECOG, Eastern Cooperative Oncology Group; EGFR, epidermal growth factor receptor

The ALTER0303 trial was the first randomized, double-blinded, placebo-controlled, Phase III study on Chinese patients with advanced or metastatic NSCLC who received at least two lines of prior chemotherapies (Fig. 1). Eligible patients were randomized 2:1 to receive either anlotinib (12 mg) or placebo once daily on the 2-week on and 1-week off schedule. Randomization was stratified by Eastern Cooperative Oncology Group (ECOG) performance status (PS) (0 vs. 1), pathologic type (squamous vs. non-squamous), and driver gene mutation status (sensitive mutation vs. wild-type).

Key inclusion criteria were as follows: (1) advanced or metastatic, pathologically diagnosed NSCLC with measurable tumor lesion by Response Evaluation Criteria In Solid Tumors (RECIST) version 1.1; (2) received at least two lines of prior chemotherapy, with wild-type epidermal growth factor receptor (EGFR) or anapastic lymphoma kinase (ALK), or sensitive mutations but failed in relevant targeted therapy; (3) ECOG 0 or 1. Key exclusion criteria were as follows: (1) hypertension with systolic blood pressure exceed 140 mmHg or diastolic blood pressure exceed 100 mmHg after treatment; (2) squamous cell carcinoma of central type with bleeding risk; (3) previous anti-angiogenesis treatment including bevacizumab or small-molecule TKIs; (4) with unknown EGFR and ALK mutation type, or sensitive mutation without receiving relevant targeted therapy; (5) with unstable central nervous system metastases. If the investigators considered that the patient continued to achieve clinical benefit but with a stable performance status and without rapid disease progression, the patient would be permitted to continue the treatment beyond the initially defined criteria of progressive disease. However, the decision to continue treatment should be discussed with the Sponsor Medical Manager and documented in the study records.

The primary endpoint was OS of modified intent-to-treat population (patients randomized and received at least one dose of anlotinib or placebo). Secondary endpoints included objective response rate (ORR), investigator-determined progression-free survival (PFS), duration of response (DoR), and quality of life (QoL) assessed using the European Organization for Research and Treatment of Cancer Quality-of-Life-Questionnaire-Core-30 (EORTC-QLQ-C30). The clinical trial registry and identifying number is NCT02388919.

The predicted median OS was 11 months for the anlotinib arm and 7 months for the placebo arm. Approximately 291 deaths were required to provide 85% power to detect a hazard ratio (HR) of 0.70 with an overall type I error of 5% at two sides. This assumes accrual of 12 months and follow-up of 12 months, with 10% lost to follow-up. To meet this statistical hypothesis, 450 patients were required with at least 291 OS events occurred. Independent Data Monitoring Committee (IDMC) was required for the analysis of primary endpoint.

OS was compared between the anlotinib and placebo arms at the interim analysis via a two-sided log-rank test stratified by ECOG PS, pathologic type, and driver gene mutation status in the ALTER0303 trial. OS curves for each arm were estimated using Kaplan–Meier product limit method. The HR and 95% confidence interval (CI) were estimated using a stratified Cox proportional hazards model.

## Review process of anlotinib

**Fig. 2:**
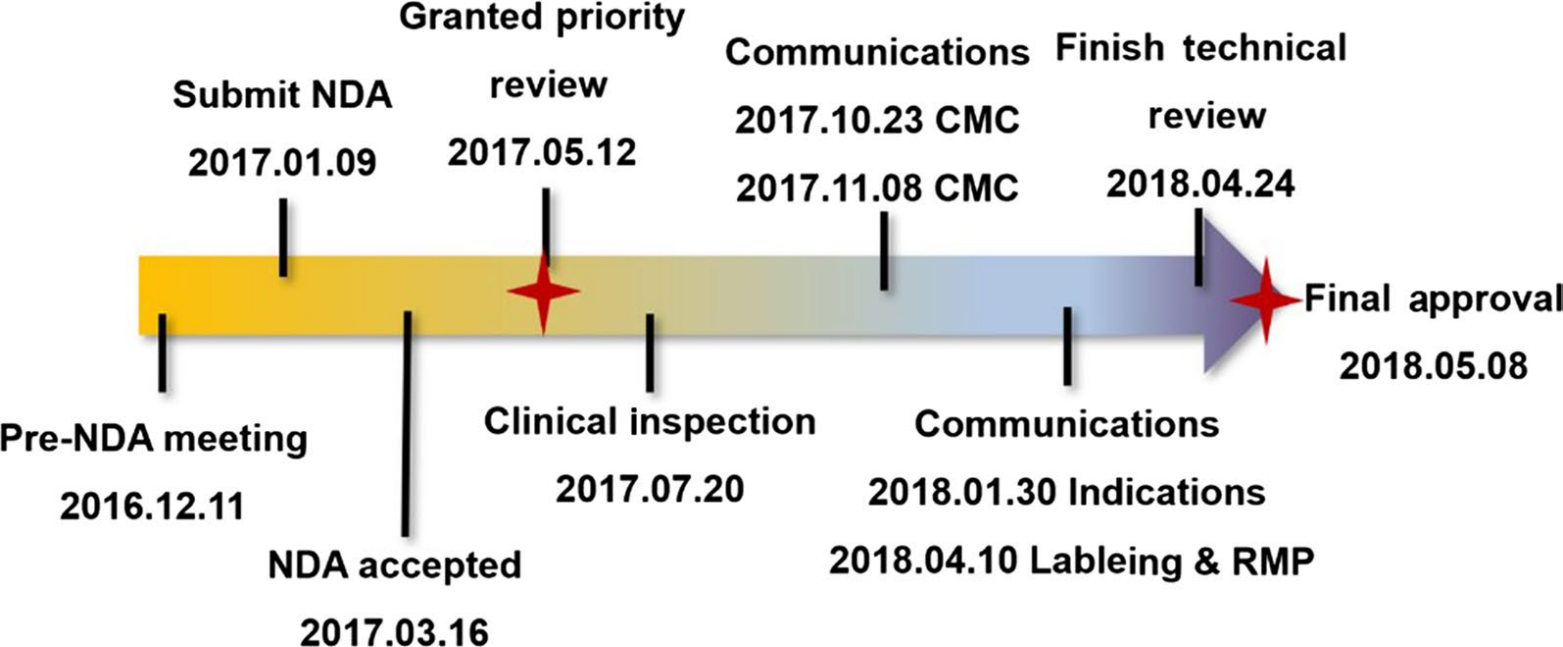
The review process of anlotinib. NDA, new drug application; CMC, chemistry manufacture control; RMP, risk management program

On November 8, 2016, IDMC suggested to stop the ALTER0303 trial recruitment according to the results of the interim analysis. Subsequently, CTTQ asked for pre-New Drug Application (NDA) meeting with China CDE on December 11, 2016. CDE agreed that the NDA package of anlotinib was acceptable based on the available data. The NDA application of anlotinib was granted as a priority review on May 12, 2017. From July 20 to 27, 2017, the Center for Drug Inspection (CDI) conducted routine inspection of the ALTER0303 trial conducted in four cancer sites in China. During the review process, CDE and CTTQ held four communication meetings, focusing on chemical manufacturing and control, indications, labeling, and risk management issues (Fig. 2).

Between March 1, 2015 and August 31, 2016, the trial screened 601 patients, randomized 439 patients, in which 437 patients receiving at least one dose of the anlotinib or placebo were included for efficacy analyses according to intent-to-treat principle. The median age was 57 (range 31–74) years in the placebo arm and 59 (range 20–75) years in the anlotinib arm. Key demographic and disease characteristics of patients were well balanced between the two arms (Table 1). The majority patients were diagnosed with stage IV metastatic disease (94.5%). The proportions of patients who have received two lines and at least three lines of chemotherapies were 56.1% and 43.0%, respectively. The proportions of patients with squamous cell carcinoma and non-squamous cell carcinoma were 19.7% and 80.3%, respectively. The proportions of patients with EGFR and ALK sensitive mutations were 31.6% and 1.6%, respectively.

**Table 1:** Baseline demographics and disease characteristics of the 437 patients with advanced non-small cell lung cancer who were enrolled in the ALTER0303 trial

Characteristic	Placebo arm [cases (%)]	Anlotinib arm [cases (%)]
Total	143	294
Gender		
Male	97 (67.8)	188 (63.9)
Female	46 (32.2)	106 (36.1)
Smoking status		
Never smoked	66 (46.2)	151 (51.4)
Light ex-smoker	67 (46.9)	130 (44.2)
Current smoker	10 (7.0)	13 (4.4)
Baseline ECOG PS		
0	22 (15.4)	59 (20.1)
1	120 (83.9)	233 (79.3)
2	1 (0.7)	2 (0.7)
Pathologic type		
Adenocarcinoma	108 (75.5)	228 (77.6)
Squamous or adenosquamous cell carcinoma	33 (23.1)	53 (18.0)
Other types	2 (1.4)	13 (4.4)
Clinical stage at screening		
IIIB	7 (4.9)	15 (5.1)
IV	136 (95.1)	277 (94.2)
Others	0	2 (0.7)
Number of metastatic site		
≤ 3	81 (56.6)	171 (58.2)
> 3	62 (43.4)	123 (41.8)
Surgery history	91 (63.6)	153 (52.0)
Pre-chemotherapy		
Two lines	78 (54.5)	167 (56.8)
Three lines or more	65 (45.5)	123 (41.8)
After first line	0	4 (1.4)
EGFR mutation status^a^		
Wild-type	98 (68.5)	201 (68.4)
Sensitive mutation	45 (31.5)	93 (31.6)
ALK status		
Positive	2 (1.4)	5 (1.7)
Negative	136 (95.1)	277 (94.2)
Unknown	5 (3.5)	12 (4.1)
Pre-TKI use^a^ in EGFR mutation patient	42 (93%)	91 (98%)

ECOG, Eastern Cooperative Oncology Group; PS, performance status; EGFR, epidermal growth factor receptor; ALK, anaplastic lymphoma kinase; TKI, tyrosine kinase inhibitor

^a^EGFR mutations include Exon 19 deletion and Exon 21 Leu858Arg. Pre-TKI use include: gefitinib, erlotinib, acotenib, afatinib and AZD9291

### Efficacy

The prespecified interim OS analysis was conducted when 292 deaths occurred (66.8% of the planned number of events for final analysis) on January 6, 2017. Per the O’Brien-Fleming boundary, the two-sided significance level for the interim OS analysis with 292 deaths was 0.005. As of the cutoff date, 93 (65.0%) patients in the placebo arm received further anti-cancer treatment including chemotherapy, targeted therapy, and traditional Chinese medicine, in which 59 (41.3%) patients received a third-line chemotherapy; 143 (49.0%) patients in the anlotinib arm received further anti-cancer treatment, in which 66 (22.6%) received further chemotherapy.

**Fig. 3:**
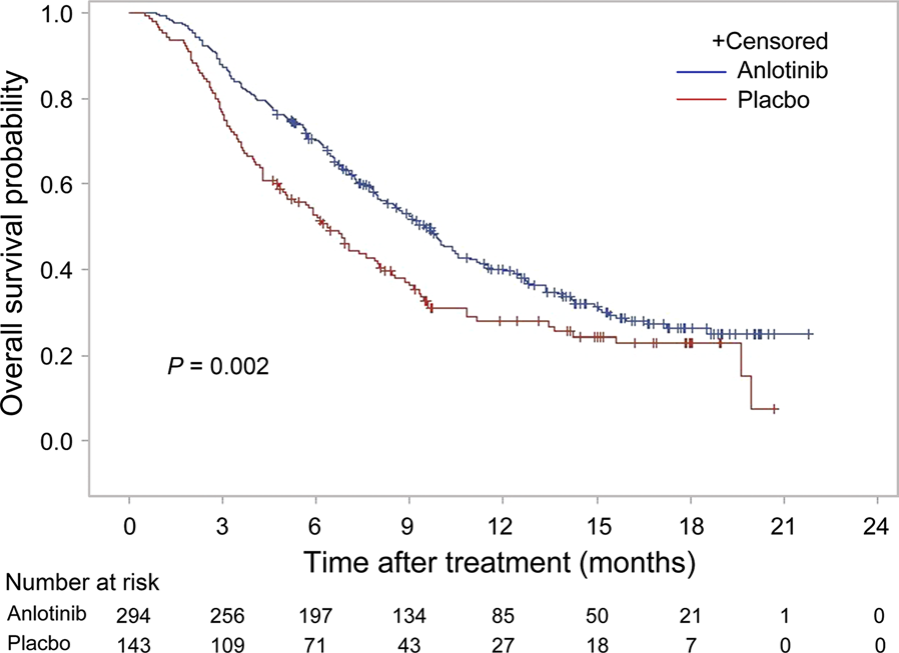
Kaplan–Meier overall survival curves of patients with advanced non-small cell lung cancer in the anlotinib and place arms

China CDI inspected all 135 case report forms (CRFs) in the anlotinib arm and 35 in the placebo arm from Shanghai Chest Hospital (Shanghai, China), Tianjin Cancer Hospital (Tianjin, China), Henan Province Cancer Hospital (Zhengzhou, Henan, China), and Linyi Cancer Hospital (Linyi, Shandong, China). The OS data of three patients (2 in the anlotinib arm and 1 in the place arm) were modified after inspection. CDE re-analyzed the OS data after the inspection of the ALTER0303 trial. Despite more further treatments in the placebo arm, there was a significance improvement in OS for patients who received anlotinib compared with placebo with a 3.1-month prolongation in median OS (HR = 0.70, 95% CI 0.55–0.89; *P* = 0.002; Table 2 and Fig. 3). Key secondary endpoint PFS was analyzed after ORR results was obtained (Table 2). Per investigator assessment according to RECIST version 1.1, the confirmed ORR was 9.2% and 0.7% with median DoR of 4.8 months in the anlotinib arm. Twenty-six of 27 responses in the anlotinib arm occurred within two cycles. At the time of the interim OS analysis, PFS per investigator assessment was examined. There was a significance improvement in PFS for patients who received anlotinib compared with placebo with a 3.9-month prolongation in median PFS (HR = 0.25, 95% CI 0.19–0.31; *P* < 0.001; Table 2). QLQ-C30 score was stable in the anlotinib arm while declined in the placebo arm.

**Table 2:** Efficacy results of the ALTER0303 trial

Endpoint	Anlotinib arm (n = 294)	Placebo arm (n = 143)
OS (months, median [range])	9.46 (8.05–10.45)	6.37 (4.93–7.98)
HR (95% CI, *P* value)	0.70 (0.55–0.89, *P* = 0.002)	
PFS (months, median [range])	5.37 (4.40–5.63)	1.40 (1.07–1.50)
HR (95% CI)	0.25 (0.19–0.31, *P* < 0.001)	
ORR (CR + PR)	9.18%	0.70%
DCR (CR + PR + SD)	80.95%	37.06%

OS, overall survival; HR, hazard ratio; CI, confidence interval; PFS, progression-free survival; ORR, objective response rate; CR, complete response; PR, partial response; SD, stable disease; DCR, disease control rate

Since the proportion of Chinese patients with EGFR mutations [19] is higher than that of Western patients [20], subgroup analyses for OS based on EGFR status were performed to assess the impact of EGFR mutation status on the effectiveness of anlotinib. As shown in Table 3, anlotinib provided more survival benefit than placebo regardless of EGFR mutation status.

**Table 3:** *EGFR* status subgroup analysis of the ALTER0303 trial

*EGFR* status	Median OS (months)		HR (95% CI)	*P* value
	Placebo arm	Anlotinib arm		
Wild-type	6.47	8.87	0.73 (0.55–0.97)	0.022
Sensitive mutations^a^	6.27	10.70	0.59 (0.38–0.94)	0.023

EGFR, epidermal growth factor receptor; OS, overall survival; HR, hazard ratio

^a^Sensitive mutations include exon 19 deletion and exon 21 Leu858Arg

### Toxicity

The primary safety data were collected from 294 patients who received anlotinib and 143 patients who received placebo (Table 4). Adverse events were assessed during treatment period and within 90 days after the last dose of anlotinib or placebo. The median treatment period was 126 days (range 5 days to 46.7+ months) in the anlotinib arm and 42 days (range 7 days to 33.2 months) in the placebo arm. Dose reductions due to ADRs occurred in 25 (8.5%) patients of the anlotinib arm and 1 (0.7%) patient of the placebo arm. Additionally, 59 (20.1%) patients in the anlotinib arm and 16 (11.2%) patients in the placebo arm had a dose delay due to ADRs. Rate of death during treatment and within 30 days after the last dose of anlotinib or placebo was 6.8% (20/294) in the anlotinib arm and 5.6% (8/143) in the placebo arm; 2 (0.7%) patients died of treatment-related hemoptysis in the anlotinib arm. Serious adverse event (SAE) occurred in 123 (41.8%) patients receiving anlotinib and 29 (20.3%) patients receiving placebo. The most frequent SAEs occurred in ≥ 2% of patients in the anlotinib arm were pulmonary infection (4.1%), hemoptysis (3.4%), respiratory failure (3.1%), and seizure (3.0%).

**Table 4:** Common grade adverse drug reactions in the anlotinib or placebo arm in the ALTER0303 trial

Adverse drug reaction	Anlotinib arm [cases (%)]		Placebo arm [cases (%)]	
	All grades	≥ 3 grade	All grades	≥ 3 grade
General disorder				
Fatigue	150 (51.0)	1 (0.3)	38 (26.6)	0
Anorexia	133 (45.2)	3 (1.0)	43 (30.1)	3 (2.1)
Weight loss	66 (22.4)	0	12 (8.4)	0
Pain	42 (14.3)	2 (0.7)	15 (10.5)	2 (1.4)
Gastrointestinal disorder				
Diarrhea	103 (35.0)	3 (1.0)	21 (14.7)	0
Oropharyngeal pain	83 (28.2)	1 (0.3)	10 (7.0)	0
Oral mucositis	68 (23.1)	3 (1.0)	4 (2.8)	0
Vomiting	63 (21.4)	1 (0.3)	19 (13.3)	0
Abdominal pain	53 (18.0)	1 (0.3)	13 (9.1)	0
Nausea	52 (17.7)	0	19 (13.3)	0
Gum pain	40 (13.6)	0	2 (1.4)	0
Respiratory, thoracic, or mediastinal disorder				
Cough	110 (37.4)	2 (0.7)	33 (23.1)	1 (0.7)
Dyspnea	90 (30.6)	6 (2.0)	32 (22.4)	7 (4.9)
Cacophonia	66 (22.4)	2 (0.7)	7 (4.9)	1 (0.7)
Hemoptysis	58 (19.7)	9 (3.1)	11 (7.7)	2 (1.4)
Sputum	49 (16.7)	2 (0.7)	16 (11.2)	1 (0.7)
Upper respiratory infection	33 (11.2)	0	3 (2.1)	0
Pneumonia	28 (9.5)	12 (4.1)	9 (6.3)	3 (2.1)
Respiratory failure	10 (3.4)	10 (3.4)	3 (2.1)	3 (2.1)
Cardiovascular disorder				
Hypertension	198 (67.3)	40 (13.6)	23 (16.1)	0
Sinus tachycardia	105 (35.7)	0	47 (32.9)	0
QTc prolongations	77 (26.2)	7 (2.4)	27 (18.9)	2 (1.4)
Skin and subcutaneous tissue disorder				
Hand–foot syndrome	128 (43.5)	11 (3.7)	13 (9.1)	0
Rash	35 (11.9)	0	11 (7.7)	1 (0.7)
Musculoskeletal and connective tissue disorder				
Chest arthralgia	54 (18.4)	1 (0.3)	17 (11.9)	3 (2.1)
Lumbar and rib pain	42 (14.3)	0	11 (7.7)	0
Limbs pain	39 (13.3)	0	16 (11.2)	1 (0.7)
Kidney and urinary system disorder				
Proteinuria	85 (28.9)	7 (2.4)	19 (13.3)	1 (0.7)
Hematuria	41 (13.9)	0	8 (5.6)	0
Urinary tract infection	33 (11.2)	0	6 (4.2)	0
Endocrine system disorder				
Hypothyroidism	57 (19.4)	1 (0.3)	5 (3.5)	0
Nervous system disorder				
Dizziness	33 (11.2)	0	13 (9.1)	0
Headache	32 (10.9)	0	5 (3.5)	0
Laboratory test abnormality				
Elevated TSH	137 (46.6)	1 (0.3)	9 (6.3)	0
Hyper triglycerides	126 (42.9)	9 (3.1)	34 (23.8)	0
Hypercholesterolemia	119 (40.5)	0	20 (14.0)	0
Hyper γ-glutamyl transferase	87 (29.6)	13 (4.4)	26 (18.2)	9 (6.3)
Hyperbilirubinemia	76 (25.9)	5 (1.7)	21 (14.7)	2 (1.4)
Hyponatremia	66 (22.4)	24 (8.2)	12 (8.4)	5 (3.5)
Hyper LDL	60 (20.4)	2 (0.7)	11 (7.7)	0
Lymphocytopenia	55 (18.7)	14 (4.8)	27 (18.9)	8 (5.6)
Hypoalbuminemia	53 (18.0)	1 (0.3)	18 (12.6)	1 (0.7)
Elevated alkaline phosphatase	48 (16.3)	7 (2.4)	18 (12.6)	4 (2.8)
Elevated alanine transaminase	46 (15.6)	2 (0.7)	13 (9.1)	0
Elevated aspartate transaminase	44 (15.0)	3 (1.0)	15 (10.5)	0
Hypophosphatemia	31 (10.5)	4 (1.4)	10 (7.0)	2 (1.4)
Hypokalemia	31 (10.5)	2 (0.7)	7 (4.9)	0
Thrombocytopenia	30 (10.2)	3 (1.0)	6 (4.2)	0
Elevated lipase	17 (5.8)	7 (2.4)	2 (1.4)	1 (0.7)

QTc, corrected QT interval; TSH, thyroid stimulating hormone; LDL, low-density lipoprotein

The most common ADRs occurred in ≥ 10% of patients in the anlotinib arm were hypertension (67.4%), hand–foot syndrome (43.5%), anorexia (45.2%), oropharyngeal pain (28.2%), and hemoptysis (19.7%). The most common laboratory test abnormalities that worsened compared with baseline levels in ≥ 25% of patients included elevated triglyceride (42.9%), cholesterol (40.5%), γ-transglutaminase (GGT, 29.6%), thyroid stimulating hormone (TSH, 46.6%) and urine protein (28.9%).

The most common ADRs included hypertension (67.4% in the anlotinib arm vs. 16.1% in the placebo arm; 13.6% with Grade 3 hypertension in the anlotinib arm vs. 0% with Grade 3 hypertension in the placebo arm), rash (12.0% in the anlotinib arm vs. 7.7% in the placebo arm; 0% with Grade 3 rash in the anlotinib arm vs. 0.7% with Grade 3 rash in the placebo arm), hand–foot syndrome (43.5% in the anlotinib arm vs. 9.1% in the placebo arm; 3.7% with Grade 3 hand–foot syndrome in the anlotinib arm vs. 0% with Grade 3 hand–foot syndrome in the placebo arm), and hemoptysis (19.7% in the anlotinib arm vs. 7.7% in the placebo arm; 3.1% with Grade 3 hemoptysis in the anlotinib arm vs. 1.4% with Grade 3 hemoptysis in the placebo arm). In addition, 77 (26.2%) patients receiving anlotinib and 27 (18.9%) patients receiving placebo experienced corrected QT interval (QTc) prolongation.

Most cases of hypertension and hand–foot syndrome occurred within 2 weeks, and was manageable under common antihypertensive drugs and urea cream.

Hemoptysis occurred in 58 (19.7%) patients in the anlotinib arm and 11 (7.7%) patients in the placebo arm; 9 (3.1%) patients in the anlotinib arm developed Grade 3–4 hemoptysis, but only 1 (0.7%) patient in the placebo arm developed Grade 3 hemoptysis. Besides, more cases of other common hemorrhagic ADRs including hemorrhinia, oulorrhagia, and occult blood were reported in the anlotinib arm comparing with those in the placebo arm. The risk of bleeding due to anti-angiogenesis TKIs was definite. Hemoptysis was the most important risk of anlotinib, considering the high rate of Grade 3 hemoptysis in the anlotinib arm, which was fetal in high-risk patients. Therefore, squamous cell carcinoma of central type and a high risk of hemoptysis were listed as contraindications of anlotinib.

QT interval prolongation was a common ADR of anti-angiogenesis TKIs such as sorafenib, sunitinib, and vandetanib [21]. In the ALTER0303 trial, QT interval prolongation occurred in 77 (26.2%) patients in the anlotinib arm and 27 (18.9%) patients in the placebo arm. Seven (2.4%) patients in the anlotinib arm experienced Grade 3 QT interval prolongation without any clinical symptoms. Routine electrocardiogram (ECG) monitoring should be conducted in clinical practice for patients receiving anlotinib.

Transient epilepsy or seizures occurred only in 3 patients in the anlotinib arm. The mechanism of nervous system toxicity is undefined and needs to be defined in future.

## Discussion

The development and approval process of anlotinib was accelerated on two aspects. Firstly, CDE participated in early development of anlotinib, considering the unmet medical needs of advanced NSCLC patients after two lines of chemotherapy. Base on the safety and efficacy data of the anlotinib arm, CDE suggested to conduct a confirmatory trial on advanced NSCLC and decided the clinical endpoints and the interim analysis. Secondly, various reforming measures taken by NMPA during review and approval accelerated the approval of anlotinib, which allow conducting clinical inspection and medical review in parallel. Considering the unmet medical needs of patients with advanced NSCLC and the efficacy results of the ALTER0303 trial, anlotinib was granted priority review, which accelerated the review and approval processes. During the review process, communication meetings focus on general technical question and answer helped to solve technical matters efficiently.

After reviewing, inspecting, and re-analyzing clinical data of anlotinib, NMPA concluded that the ALTER0303 trial was conducted in accordance with good clinical practice guidelines and other applicable Chinese regulatory requirements. The major deficiency in study design was using placebo control other than physician’s choice or best supportive care as the control measure. Despite this trial deficiency, we consider that the study design of the ALTER0303 trial followed the basic science and the current clinical practices in China, the primary endpoint OS is a golden standard for the efficacy assessment of anti-cancer agents, and the usage of placebo as a third-line treatment in the ALTER0303 trial was acceptable when anlotinib was under development. The usage of anlotinib in patients with advanced NSCLC who have progressed on at least two lines of chemotherapy resulted in a significant OS prolongation compared with placebo. The anlotinib arm demonstrated a 3.1-month prolongation in median OS compared with the placebo arm, with the risk of death decreased by 30%. The improvement in OS was statistically significant and clinically meaningful.

Several other anti-angiogenesis TKIs failed to bring survival benefit in NSCLC patients. For example, TKIs alone or in combination with chemotherapies offered patients little to no benefit, with significant toxicity [14–16]. The study design of the ALTER0303 trial is similar with that of the MISSHION trial [14]. However, the ALTER0303 trial achieved its primary endpoint. Firstly, anlotinib selectively inhibits the kinase activities of VEGFR1-3, in addition to other angiogenic and oncogenic pathway-related kinases, including FGFR1-4, PDGFRα/β, especially RET and KIT. We considered that multiple targets beyond angiogenic kinases helped anlotinib to achieve higher anti-cancer efficacy. The response rate of patients treating with single-agent anlotinib was 9.2%, which supported the multiple-target opinion. Secondly, hypertension and hemorrhage were the most common ADRs of anlotinib and leaded to drug withdraw. Thus, the ALTER0303 trial excluded patients with unmanageable hypertension and squamous cell carcinoma of central type so that patients could tolerate longer duration of anlotinib treatment to achieve more clinical benefits. Finally, placebo control was the design deficiency but it helped to detect the absolute efficacy of anlotinib.

The indication of anlotinib is patients with EGFR sensitive mutation, which was determined after the communication with the primary investigator of the ALTER0303 trial and CTTQ during the NDA review process in January 2018. CDE made this decision based on the following considerations. Firstly, negligible amounts of patients with lung adenocarcinoma were included in the ALTER0303 trial, of which 138 (31.6%) patients had EGFR sensitive mutation, and 133 (96%) received prior EGFR TKI and at least two lines of systemic chemotherapy. When the ALTER0303 trial was initiated, osimertinib (AZD9291), the third-generation EGFR TKI, had not been approved by CFDA. EGFR T790M mutation detection was therefore not essential. Secondly, which is the most important, patients with EGFR mutation benefited from anlotinib treatment comparing with placebo, with the median OS prolongation of 4.4-month (HR = 0.59, 95% CI 0.38–0.94, *P* = 0.023). Post-hoc analysis showed that 18 patients with EGFR TKI resistance were confirmed to have EGFR exon 20 T790M mutation, in which only one received AZD9291 after treatment failure of erlotinib. The median OS was 21.5 months among patients with T790M who received anlotinib, but was 6.6 months among patients who received placebo notwithstanding the limited sample size. CDE suggested CTTQ to pay more attention to the biomarker-associated eligibility criteria, and the control treatment choice should be more prospective and considering about the changing of clinical practices. CTTQ promised to conduct prospective clinical trials to confirm the clinical benefit of anlotinib in patients with different EGFR T790M statuses in the future.

During the NDA assessment of anlotinib, the medical group system reviewed the previous phase 3 clinical trials of anti-angiogenesis TKIs for advanced NSCLC. In our opinion, the ALTER0303 trial has demonstrated outstanding efficacy and manageable toxicity profiles of anlotinib and is a milestone in the research on third-line single agent for NSCLC, and it provided valuable experiences in the study design of anti-angiogenesis TKIs in the future. CDE considers the patients’ clinical benefits the most important. Only if the drug demonstrates substantial improvement on a clinically significant endpoint over available therapies under serious conditions, CDE would like to accept surrogate endpoint, single-arm trial, or conditionally approval new drug based on a Phase II trial, then completely approve after a confirmatory trial. CDE encourages communications during clinical development. The following questions or recommendations should be considered while design a confirmatory trial of anti-angiogenesis TKI.

Whether the indication definition or main eligible criteria is appropriate? Could the trial design fit the medical need both during the research period and at the time of NDA review?

Could monotherapy guarantee the efficacy? If choosing monotherapy, please consider the rationalization of the dosage.

Is there solid rational for combination or add on design? For example, combine with an immune checkpoint inhibitor. Is there solid mechanism or promising clinical evidence to support such combination? Whether anti-angiogenesis TKIs could improve immune microenvironment which could generate synergistic effect with immune checkpoint inhibitors. The tolerance and safety profile of combination should also be considered, for example, cardiac toxic events might increase under such combination.

Is the primary endpoint of clinical trial appropriate? Could the statistical hypothesis and statistical analysis plan guarantee both type I error and clinical significance? At present, OS is considered a golden standard of primary endpoint in anti-cancer trials, and appropriate surrogate endpoints such as PFS are widely used as primary endpoints in randomized trials. ORR has become a surrogate endpoint in single-arm trials for accelerated approval. Please ensure that the endpoint fit the efficacy assessment and indication definition of anti-angiogenesis TKIs.

No effect of predictive biomarker has been verified in current anti-angiogenesis therapies for different solid tumors. However, we still consider it is worthy of attention to explore potential predictive biomarkers in the future to improve the efficiency of clinical development of anti-angiogenesis therapies.

The toxicity profile of anlotinib is moderate compared with placebo, considering 3.1-month survival benefit in the ALTER0303 trial. However, more attention should be paid to certain hemoptysis events, and the uncertain nervous toxicity of anlotinib should be observed and verified in ongoing trials. NMPA also requested that CTTQ Pharma conduct further research on risk management beyond the ongoing registry trials of anlotinib in extensive stage small cell lung cancer and metastatic soft tissue sarcoma. Lung cancer is with high heterogeneity and complicated symptoms. Thus, the dosage selection is the key scientific issue during early development. The Phase I trial of anlotinib had shown unacceptable drug accumulation, hypertension, and hand and foot syndrome on the dosage of 10 mg once daily. Learning the international experience in developing anticancer therapies, the dosage of anlotinib was determined as 12 mg on a 2-week on and 1-week off schedule. The dosage was confirmed in further trials accompany with acceptable toxicity and compliance. However, the dosage of anlotinib may need to be optimized in future trials. Improvement of symptoms, which include patient-reported outcomes, quality of life, and remission of cancer-related cough and pain, was considered a direct clinical benefit. CDE encouraged the development of symptomatic endpoints in registered trial in the future.

## Conclusions

Anlotinib demonstrated a clinically significant OS prolongation as a novel therapeutic option for advanced or metastatic NSCLC following at least two lines of chemotherapy. The design and implementation of the ALTER0303 trial are with defect but followed basic scientific research guidelines to guarantee the data quality. Anlotinib met the unmet medical need at present, even at the background that first immune checkpoint inhibitor nivolumab was approved as a second-line treatment of NSCLC in June 2018.

## Data Availability

The clinical data that support the conclusions of this review were submitted by Chia Tai Tianqing Pharmaceutical Group Co., Ltd, reviewed and inspected by Center for Drug Evaluation and Center for Drug Inspection of China National Medical Products Administration. The public report was released on the website of the CDE information disclosure section (http://www.cde.org.cn) in August 2018.

## Abbreviations

NSCLC: non-small cell lung cancer
OS: overall survival
TKI: tyrosine kinase inhibitor
NMPA: National Medical Products Administration
CFDA: China Food and Drug Administration
EMA: European Medicines Agency
VEGFR: vascular endothelial growth factor receptor
PDGFR: platelet-derived growth factor receptor
FGFR: fibroblast growth factor receptor
U.S. FDA: United States Food and Drug Administration
CTTQ: Chia Tai Tianqing
RET: rearranged during transfection
DLT: dose-limiting toxicity
CDE: Center for Drug Evaluation
ECOG: Eastern Cooperative Oncology Group
RECIST: Response Evaluation Criteria In Solid Tumor
EGFR: epidermal growth factor receptor
SBP: systolic blood pressure
DBP: diastolic blood pressure
CNS: central nervous system
ALK: anapastic lymphoma kinase
mITT: modified intent to treat
ORR: objective response rate
PFS: progression-free survival
DoR: duration of response
QoL: quality of life
EORTC-QLQ-C30: European Organization for Research and Treatment of Cancer Quality-of-Life-Questionnaire-Core-30
HR: hazard ratio
IDMC: independent data monitoring committee
CI: confidence interval
IDMC: Independent Data Monitoring Committee
NDA: New Drug Application
CDI: Center for Drug Inspection
ITT: intent-to-treat
CRF: case report form
ADR: adverse drug reaction
SAE: serious adverse event
GGT: γ-transglutaminase
TSH: thyroid stimulating hormone
TB: total bilirubin
QTc: corrected QT interval
